# H9N2 Influenza A Virus Isolated from a Greater White-Fronted Wild Goose (*Anser albifrons*) in Alaska Has a Mutation in the PB2 Gene, Which Is Associated with Pathogenicity in Human Pandemic 2009 H1N1

**DOI:** 10.1128/genomeA.00869-16

**Published:** 2016-09-01

**Authors:** Andrew B. Reeves, Hon S. Ip

**Affiliations:** a U.S. Geological Survey, Alaska Science Center, Anchorage, Alaska, USA; b U.S. Geological Survey, National Wildlife Health Center, Madison, Wisconsin, USA

## Abstract

We report here the genomic sequence of an H9N2 influenza A virus [A/greater white-fronted goose/Alaska/81081/2008 (H9N2)]. This virus shares ≥99.8% identity with a previously reported virus. Both strains contain a G590S mutation in the polymerase basic 2 (PB2) gene, which is a pathogenicity marker in the pandemic 2009 H1N1 virus when combined with R591.

## GENOME ANNOUNCEMENT

Avian influenza A viruses (AIV) of the H9N2 subtype are of interest due to their potential to cause or contribute to pandemics in domestic birds and infect humans (1, 2). Recently, Hussein et al. (3) reported an AIV of the H9N2 subtype collected from a wild duck [A/northern shoveler/Interior Alaska/8BM3470/2008 (H9N2), here AK08]. This virus contained the G590S mutation in the polymerase basic (PB) 2 gene, which is associated with infections in humans (3, 4). Hussein et al. (3) demonstrated that the introduction of a point mutation (Q591K) increased polymerase activity and allowed the virus to replicate in human cells. As part of an interagency program for early detection of highly pathogenic H5N1 AIV, wild birds were sampled across North America beginning in 2006 (5). We sequenced an AIV genome collected from a wild goose near the community of Point Hope, Alaska, in 2008 using an Illumina MiSeq platform. Sequence assembly and computational analyses were performed with Geneious R9. This virus, A/greater white-fronted goose/Alaska/81081/2008 (H9N2) (here 81081), shared ≥99.8% nucleotide identity with AK08 across all eight gene segments (PB2, 99.96%; PB1, 99.82%; polymerase acidic [PA], 99.91%; hemagglutinin [HA], 99.76%; nucleoprotein [NP], 99.93%; neuraminidase [NA], 99.93%; matrix [MA], 99.80%; nonstructural [NS], 99.88%). In total, 16 point mutations were observed between the open reading frames (ORFs) of the two viruses, with 10 synonymous and six nonsynonymous site substitutions. None of the four mutations found in the PB2 ORF were predicted to result in an amino acid substitution, nor did they occur in any of the residues highlighted in AK08 (3).

Although both viruses were collected in Alaska, the distance between Point Hope and Minto Flats State Game Refuge, the sampling location for AK08, is approximately 878 km. In addition, the original virus sample for 81081 was collected on 22 May 2008, while the AK08 sample was collected on 1 September of that same year. Thus, these two viruses provide another example of H9N2 AIVs demonstrating genomic constellation integrity over large geographic distances and time, similar to the results of Lee et al. (6) and Ramey et al. (7). Last, multiple segment sequences of AK08 were closely related genetically and temporally to sequences of AIVs collected in both Asia and North America (3). Our finding of a virus with a highly similar genome provides further evidence for the intercontinental exchange of AIVs between Eurasia and North America and particularly high detection of evidence for interhemispheric exchange in viral genomes of the H9N2 subtype (8–10).

### Accession number(s).

The GenBank accession numbers for the complete sequence of A/greater white-fronted goose/81081/2008(H9N2) are KX377327 to KX377334.

## References

[1] Fusaro A, Monne I, Salviato A, Valastro V, Schivo A, Amarin NM, Gonzalez C, Ismail MM, Al-Ankari A-R, Al-Blowi MH, Khan OA, Maken Ali AS, Hedayati A, Garcia Garcia J, Ziay GM, Shoushtari A, Al Qahtani KN, Capua I, Holmes EC, Cattoli G 2011 Phylogeography and evolutionary history of reassortant H9N2 viruses with potential human health implications. J Virol 85:8413–8421. doi:10.1128/JVI.00219-11. 21680519 PMC3147996

[2] World Health Organization 2016 Influenza at the human-animal interface: summary and assessment. World Health Organization, Geneva, Switzerland http://www.who.int/influenza/human_animal_interface/en/.

[3] Hussein IT, Ma EJ, Hill NJ, Meixell BW, Lindberg M, Albrecht RA, Bahl J, Runstadler JA 2016 A point mutation in the polymerase protein PB2 allows a reassortant H9N2 influenza isolate of wild-bird origin to replicate in human cells. Infect Genet Evol 41:279–288. doi:10.1016/j.meegid.2016.04.011. 27101787 PMC4868792

[4] Mehle A, Doudna JA 2009 Adaptive strategies of the influenza virus polymerase for replication in humans. Proc Natl Acad Sci USA 106:21312–21316. doi:10.1073/pnas.0911915106. 19995968 PMC2789757

[5] Ip HS, Flint PL, Franson JC, Dusek RJ, Derksen DV, Gill RE Jr, Ely CR, Pearce JM, Lanctot RB, Matsuoka SM, Irons DB, Fischer JB, Oates RM, Petersen MR, Fondell TF, Rocque DA, Pedersen JC, Rothe TC 2008 Prevalence of influenza A viruses in wild migratory birds in Alaska: patterns of variation in detection at a crossroads of intercontinental flyways. Virol J 5:71. doi:10.1186/1743-422X-5-71. 18533040 PMC2435106

[6] Lee DH, Park JK, Yuk SS, Erdene-Ochir TO, Kwon JH, Lee JB, Park SY, Choi IS, Lee SW, Song CS 2014 Complete genome sequence of a natural reassortant H9N2 avian influenza virus found in bean goose (*Anser fabalis*): direct evidence for virus exchange between Korea and China via wild birds. Infect Genet Evol 26:250–254. doi:10.1016/j.meegid.2014.06.007. 24953505

[7] Ramey AM, Reeves AB, Sonsthagen SA, TeSlaa JL, Nashold S, Donnelly T, Casler B, Hall JS 2015 Dispersal of H9N2 influenza A viruses between East Asia and North America by wild birds. Virology 482:79–83. doi:10.1016/j.virol.2015.03.028. 25827532

[8] Bahl J, Pham TT, Hill NJ, Hussein IT, Ma EJ, Easterday BC, Halpin RA, Stockwell TB, Wentworth DE, Kayali G, Krauss S, Schultz-Cherry S, Webster RG, Webby RJ, Swartz MD, Smith GJ, Runstadler JA 2016 Ecosystem interactions underlie the spread of avian influenza A viruses with pandemic potential. PLoS Pathog 12:e1005620. doi:10.1371/journal.ppat.1005620. 27166585 PMC4864295

[9] Dong G, Luo J, Zhang H, Wang C, Duan M, Deliberto TJ, Nolte DL, Ji G, He H 2011 Phylogenetic diversity and genotypical complexity of H9N2 influenza A viruses revealed by genomic sequence analysis. PLoS One 6:e17212. doi:10.1371/journal.pone.0017212. 21386964 PMC3046171

[10] Zhu Y, Hu S, Bai T, Yang L, Zhao X, Zhu W, Huang Y, Deng Z, Zhang H, Bai Z, Yu M, Huang J, Shu Y 2014 Phylogenetic and antigenic characterization of reassortant H9N2 avian influenza viruses isolated from wild waterfowl in the East Dongting Lake wetland in 2011–2012. Virol J 11:77. doi:10.1186/1743-422X-11-77. 24779444 PMC4012091

